# Spectrophotometric Methods for Determination of Sunitinib in Pharmaceutical Dosage Forms Based on Ion-pair Complex Formation

**DOI:** 10.22037/ijpr.2020.1101119

**Published:** 2020

**Authors:** Effat Souri, Eynollah Amoon, Nazanin Shabani Ravari, Fereshteh Keyghobadi, Maliheh Barazandeh Tehrani

**Affiliations:** a *Department of Medicinal Chemistry, Faculty of Pharmacy, Tehran University of Medical Sciences, Tehran, Iran. *; b *Department of Pharmaceutics, Faculty of Pharmacy, Tehran University of Medical Sciences, Tehran, Iran.*

**Keywords:** Sunitinib, Bromocresol purple, Bromothymol blue, Bromophenol blue, Ion-pair complex formation

## Abstract

Three rapid spectrophotometric methods were developed for the determination of sunitinib based on the formation of ion-pair complex in acidic medium with bromocresol purple, bromothymol blue, and bromophenol blue. The formed ion-pair complexes, extractable with chloroform, were measured at 422 nm for bromocresol purple, 425 nm for bromothymol blue and 427 nm for bromophenol blue. All these methods were optimized for the pH of buffer and the volume of the reagent. The methods were linear over the range of 1-200 µg/mL for bromocresol purple, 1-150 µg/mL for bromothymol blue, and 2-200 µg/mL for bromophenol blue with a very low limit of quantification and acceptable accuracy and precision. Using the proposed methods for determination of sunitinib in pharmaceutical dosage forms showed reliable results comparable to previously published method.

## Introduction

**Figure1:**
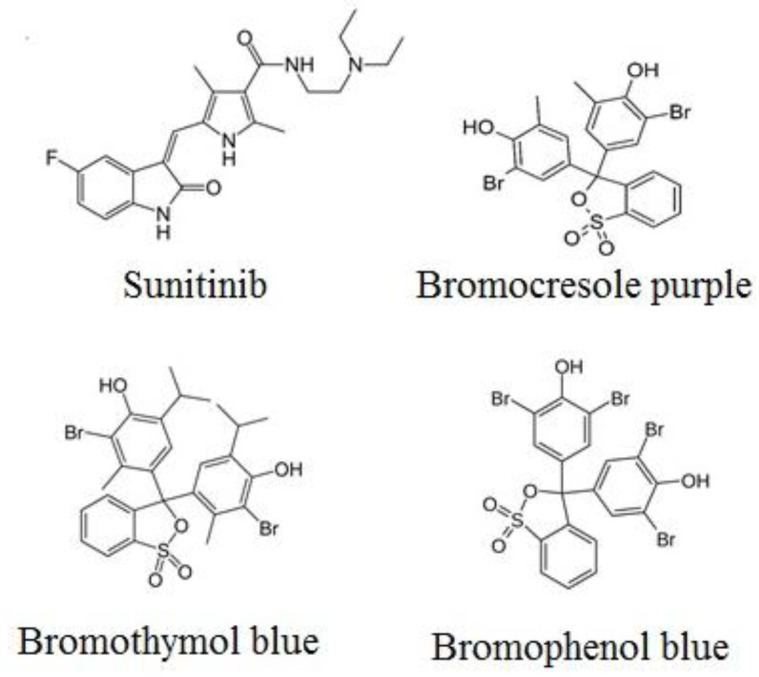
Chemical structure of sunitinib, BCP, BTB, and BPB

Sunitinib malate, (N-(2-diethylaminoethyl)-5-[(Z)-(5-fluro-2-oxo-1H-indol-3-yildene) methyl]-2,4-dimethyl-1H-pyrrole-3-carboxamide), is a broad-spectrum anticancer drug (Figure 1). Sunitinib is an orally active multi-targeted tyrosine kinase inhibitor which is effective against vascular endothelial growth factor receptor (VEGFR), platelet-p derived growth factor receptor (PDGFR), stem cell factor receptor (c-KIT) and FMS-related tyrosine kinase 3 (FLT-3) (1, 2). Blocking of these protein kinase receptors lead to inhibition of angiogenesis, proliferation, and metastasis (3-5). Sunitinib is considered as a treatment in gastrointestinal stromal tumor cells and metastatic renal cell carcinoma (6, 7). It can also prevent the growth of some other solid tumor cells such as breast, neuroendocrine hepatocellular carcinoma, pancreatic, colorectal, prostate, and non-small cell lung cancer (4, 8). 

Several analytical methods based on LC/MS/MS were reported before for the quantification of sunitinib in biological matrices (9-15). High performance liquid chromatography (HPLC) with UV detection was also reported for determination of sunitinib in human plasma (16, 17). 

Up to now, based on our literature review no report has been published for spectrophotometric measurement of sunitinib in bulk drug and pharmaceutical dosage forms. There is no need for a highly sensitive method for determination of drug in pharmaceutical dosage forms. Spectrophotometric methods are convenient and suitable substitute for other expensive methods which could be used in quality control laboratories. Therefore, the aim of the present work was to develop simple, sensitive, and reliable analytical method for determination of sunitinib in bulk and pharmaceutical dosage forms based on ion-pair complex formation. In this regard, bromocresol purple (BCP), bromothymol blue (BTB), and bromophenol blue (BPB) were used as complexing reagents (Figure 1).

## Experimental

*Chemicals*

Sunitinib malate and commercial 50 mg sunitinib capsules were kindly provided by Parsian Pharmaceuticals Co., Iran. Bromocresol purple (BCP), bromothymol blue (BTB), and bromophenol blue (BPB) were purchased from Merck (Darmstadt, Germany). Analytical grade chloroform was purchased from Merck (Darmstadt, Germany). 

*Instrumentation*

A Shimadzu double beam UV-visible spectrophotometer (UV-160A) was used for spectrophotometric measurements. The fixed bandwidth was 2 nm and 1 cm quartz cells were used. 

*Standard solutions*

By dissolving 26.6 mg of sunitinib malate in 100 mL of distilled water, a stock standard solution (5 × 10^-4^ M) was prepared. Standard solution of the reagents (5 × 10^-4^ M) were prepared by dissolving 27 mg of BCP, 31.2 mg of BTB, or 33.5 mg of BPB in 100 mL of distilled water. The phosphate buffer (0.1 M) in the pH range of 1.5-3.5 (1.5, 2.0, 2.5, 3.0, and 3.5) was prepared by dissolving appropriate amount of NaH_2_PO_4_ in distilled water and adjusting the pH value.

*General procedure*

One milliliter of sunitinib standard solution was transferred into a 100 mL separating funnel. After addition of 2 mL of phosphate buffer (pH 2.0), and 3.0 mL of BCP or 3.0 mL of BTB or 2.0 mL of BPB solution, the solution was mixed for 30 s. The resulting ion-pair complex was extracted three times by 5, 3, and 2 mL of chloroform. The organic layer was transferred into a 10 mL volumetric flask after passing through anhydrous sodium sulfate. The absorbance of the solution was measured at 422 nm for BCP, 425 nm for BTB, and 427 nm for BPB after making up the volume to 10 mL with chloroform against the proper blank reagent. Due to its susceptibility to light, the experiments were performed under controlled conditions to have minimal light exposure because of the sensitivity of sunitinib. 

*Optimization of ion-pair complex formation*

The optimum conditions and the influence of some variables affecting the ion-pair complex formation to achieve maximum absorbance were studied for the three reagents. 

*Effect of pH value*

The general procedure by using phosphate buffer at different pH values (1.5, 2.0, 2.5, 3.0, and 3.5) were performed to find out the effect of pH on ion-pair complex formation.

*Effect of reagent volume*

 Different volumes of the reagent solutions (BCP, BTP, and BPB) in the range of 0.5-4.0 mL (0.5, 1.0, 1.5, 2.0, 2.5, 3.0, 3.5, and 4.0 mL) were added to a standard solution of sunitinib malate. The mixture was treated according to the general procedure and the optimum amount of the reagent solution was found.

*Stoichiometry of the ion-pair complex formation*

 The Job’s continuous variation method was used to find out the stoichiometry of the ion-pair complex formation of sunitinib and each of the reagents. Series of sunitinib solution (1 × 10^-5^) and reagent solutions (1 × 10^-5^) were mixed in different proportions (0:10, 1:9, 2:8, 3:7, 4:6, 5:5, 6:4, 7:3, 8:4, 9:1, and 10:0) in a fixed volume of 10 mL and treated based on the general procedure. The absorbance of the extracted solutions was plotted over the mole ratio of sunitinib to study the stoichiometry of the ion-pair complex formation.

*Linearity*

Sunitinib malate calibration solutions were prepared in six series in the concentration range of 1-200 µg/mL for BCP, 1-150 µg/mL for BTB, and 2-200 µg/mL for BPB by appropriate dilution of the stock standard solution of sunitinib. All solutions were treated according to the general procedure, the calibration curves were constructed and statistical data calculated.

*Accuracy and precision*

The low, medium, and high concentration quality control solutions of sunitinib malate in the calibration range were analyzed in triplicate in one day and three consecutive days to evaluate the within-day and between-day accuracy and precision of the method. 

*Application of the proposed methods*

The content of 10 capsules was emptied and grounded in a mortar and pestle. Powdered drug equivalent to 50 mg of sunitinib (one capsule) was taken in a 50 mL volumetric flask and sonicated for 10 min with 40 mL of distilled water in an ultrasonic bath. The mixture was diluted to the volume with water and filtered. The resulting solution was determined based on the general procedure to find out the amount of sunitinib. Also, a previously reported HPLC method was applied for determination of sunitinib in dosages forms.

*Relative recovery*

The standard addition method was used for evaluation of the relative recovery of proposed methods. Standard sunitinib solution was added to a determined solution of capsule and after performing the reaction, the absorbance of the final solutions was compared to evaluate the relative recovery.

**Figure 2:**
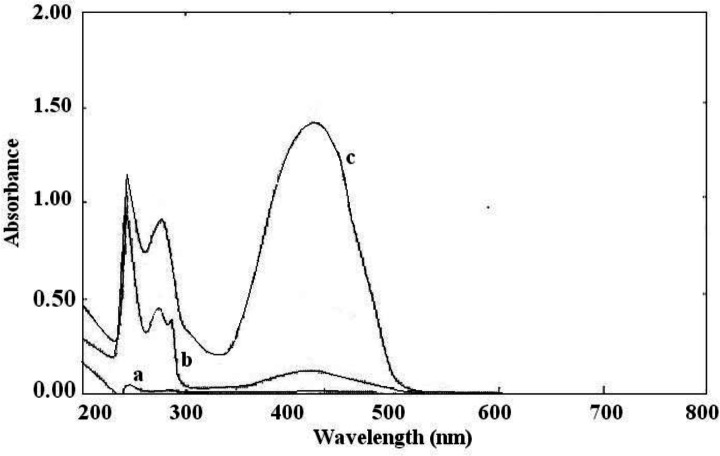
Absorption spectra of (a) sunitinib malate, (b) BCP, and (c) the ion-pair complex of sunitinib and BCP

**Figure 3:**
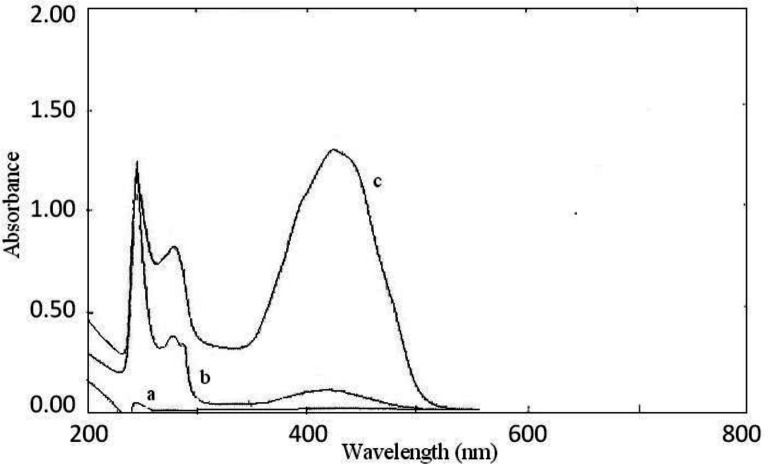
Absorption spectra of: (a) sunitinib malate, (b) BTB, and (c) the ion-pair complex of sunitinib and BTB

**Figure 4:**
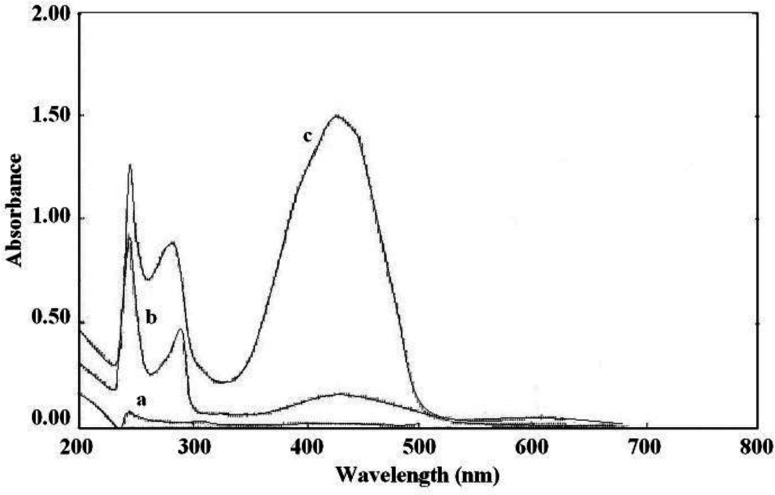
Absorption spectra of: (a) sunitinib malate, (b) BPB, and (c) the ion-pair complex of sunitinib and BPB

**Figure 5:**
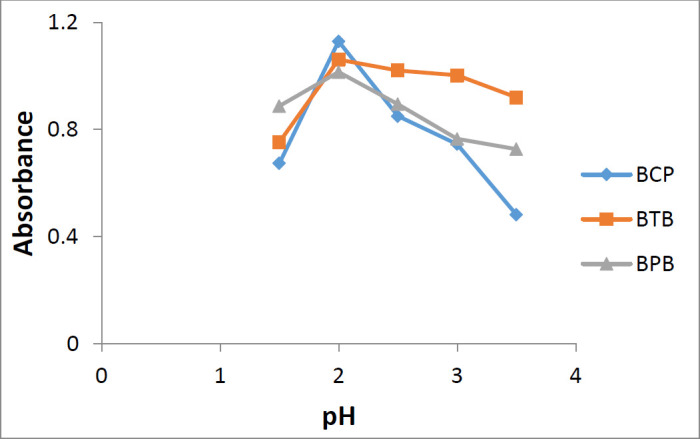
The effect of pH of the phosphate buffer on the ion-pair complex formation of sunitinib-BCP, sunitinib-BTB, and sunitinib-BPB

**Figure 6:**
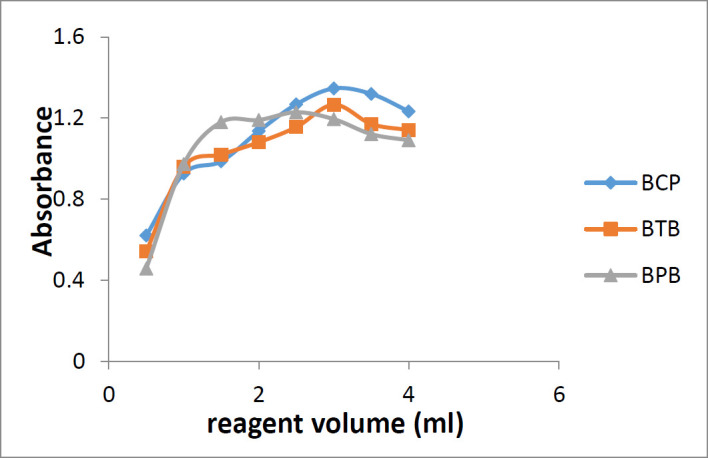
Effect of reagent volume on the formation of sunitinib-BCP, sunitinib-BTB, and sunitinib-BPB

**Figure 7:**
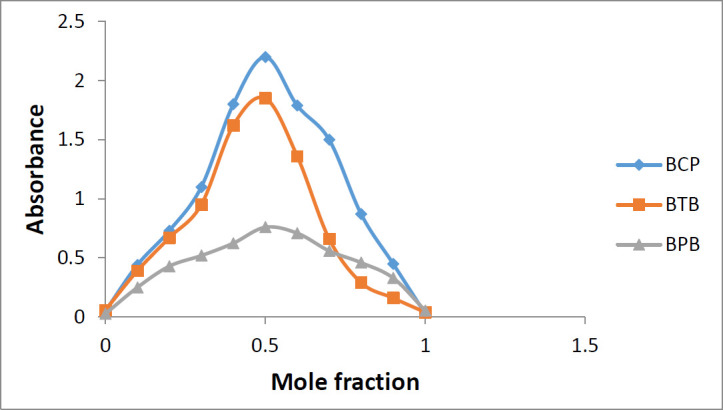
Stoichiometry of the ion-pair complex of sunitinib-BCP, sunitinib-BTB, and sunitinib-BPB

**Table 1:** Statistical data of calibration curves of sunitinib malate in standard solutions (n = 6).

**Parameters**	**BCP method**	**BTB method**	**BPB method**
Linearity range	1-200 g/mL	1-150 g/mL	2-200 g/mL
Regression equation	Y = 0.005 X + 0.045	Y = 0.005 X + 0.046	Y = 0.005 X + 0.013
SD of slope	5.2 × 10^-5^	6.3 × 10^-5^	5.5 × 10^-5^
RSD of slope (%)	1.04	1.26	1.10
SD of intercept	0.004	0.004	0.003
Correlation coefficient (r^2^)	0.997	0.999	0.998

**Table 2:** Precision and accuracy of the method for determination of sunitinib malate in standard solutions (n = 9; 3 sets for 3 days).

**Concentration added** **(g/mL)**	**Within-day (n = 3)**			**Between-day (n = 9)**		
	**Found** **(g/mL)**	**CV (%)**	**Error (%)**	**Found** **(g/mL)**	**CV (%)**	**Error (%)**
BCP method						
2.00 50.00 200.00	1.95 0.11 50.25 0.76 201.70 2.18	5.64 1.51 1.08	-2.50 0.50 0.85	1.97 0.10 50.06 0.61 200.71 1.53	5.08 1.22 0.76	-1.50 0.12 0.36
BPB method						
2.00 50.00 200.00	1.99 0.12 49.85 0.31 200.12 3.80	6.03 0.62 1.89	-0.50 -0.30 0.06	1.99 0.10 50.16 0.68 199.19 2.78	5.03 1.36 1.40	-0.50 0.32 -0.41
BTB method						
5.00 50.00 150.00	5.03 0.31 49.43 0.50 149.70 1.93	6.16 1.01 1.29	0.60 -1.14 -0.20	4.94 0.22 50.48 0.86 149.57 1.74	4.45 1.70 1.16	-1.20 0.96 -0.29

**Table 3:** Comparison of the developed methods with the reference method for the determination of Sunitinib capsules

**Method**	**Label claimed (mg)**	**Found (mean ± sd** ^*^ **)**
BCP method	50.00	50.84 ± 0.47
BTB method	50.00	51.47 ± 0.53
BPB method	50.00	51.41 ± 0.22
Reference HPLC method	50.00	51.01 ± 0.52

^*^Standard Deviation.

## Results and Discussion

*Spectrophotometric measurements*

The interaction between the reagents (BCP, BTB, and BPB) as the electron donor and the protonated amine of sunitinib as the electron acceptor formed an intensely colored charge-transfer complex, which absorbs light in the visible region. All ion-pair complexes of sunitinib with reagents showed a yellow color. The blank solution of reagents did not show significant absorbance in the range of 300-400 nm. On the other, hand intense absorbance was observed after ion-pair complex formation. The maximum absorption wavelengths were 422 nm, 425 nm, and 427 nm for BCP, BTB, and BPB, respectively (Figures 2-4). To minimize the absorbance of the reagent, the ion-pair complex solutions were measured versus a blank solution of each reagent.

*Optimization of ion-pair complex formation*

*Effect of pH value*

Using phosphate buffer in the range of 1.5-3.5, it was demonstrated that maximum complex formation and maximum absorbance was achieved at pH value of 2.0 for all three reagents (Figure 5). 

*Effect of reagent volume*

For maximum ion-pair complex formation, different amounts of reagents in the range of 0.5-4.0 mL were used. It was observed that 3 mL of BCP or BTB and 2 mL of BPB were sufficient for maximum ion-pair complex formation and maximum absorbance (Figure 6). Increasing the volume of the reagent solution did not show significant effect on the reaction yield. 

*Effect of extracting solvent*

Different solvents (chloroform, ethyl acetate, dichloromethane and diethyl ether) were used to find out the best solvent. Chloroform showed maximum extraction of the complex with no significant extraction of the reagent in the same conditions.

*Effect of time*

The effect of time on ion-pair complex formation was studied. Immediately after mixing, maximum absorbance was observed which was relatively constant at least for 72 h for all three reagents.

*Stoichiometry of the ion-pair complex formation*

Using the Job’s method of continuous variations and plotting the absorbance of the resulting solutions over the mole fraction of sunitinib malate, the stoichiometry of the complex formation was found out. As it is evident from Figure 7, the change in slope at mole fraction equal to 0.5, proves that the stoichiometry of the reaction is 1:1 (reagent:drug). 

*Linearity*

Six standard calibration curves were linear over the concentration values in the ranges stated in Table 1. The statistical data for six calibration curves are also summarized in Table 1.

*Accuracy and precision*

Three replicate analyses of selected sunitinib malate concentration levels were performed in one day and three consecutive days using the general procedure to evaluate the within-day and between-day accuracy and precision. The results are summarized in Table 2.

*Relative recovery*

The relative recovery of sunitinib malate in three proposed spectrophotometric methods was in the range of 100.34-101.48 (101.48 ± 0.09 for BCP, 100.34 ± 0.51 for BTB, and 101.24 ± 0.82 for BPB). The satisfactory recovery of sunitinib suggested that there is no significant interference from the excipients. 

*Application of the proposed methods*

The proposed spectrophotometric methods were applied for assay determination of sunitinib malate in pharmaceutical dosage form. The results are summarized in Table 3. As observed in Table 3, the results of all three methods are comparable with reference HPLC method with no significant differences.

## Conclusion

This is the first reported spectrophotometric method for the determination of sunitinib in pharmaceutical dosage forms. The proposed methods are based on the ion-pair complex formation with bromocresol purple, bromothymol blue, and bromophenol blue which are very simple, cost effective, and accurate enough for assay determination of sunitinib in pharmaceutical dosage forms.
